# Fiberoptic endoscopy evaluation of swallowing in patients with amyotrophic lateral sclerosis

**DOI:** 10.5935/1808-8694.20130061

**Published:** 2015-10-04

**Authors:** Fabiana Gonçalez D’Ottaviano, Tarcisio Aguiar Linhares Filho, Helen Maia Tavares de Andrade, Percilia Cardoso Lopes Alves, Maria Sheila Guimarães Rocha

**Affiliations:** a MSc in Otorhinolaryngology at the Santa Casa de Misericórdia de São Paulo de São Paulo (Preceptor in the ENT Residency Program of the Santa Marcelina Hospital and Clinic Otorhinus).; b MD, ENT, Specialist in ENT at the Brazilian Association of ENT-HNS.; c Head Neurologist in the Neuromuscular Disease Clinic of the Santa Marcelina Hospital (Preceptor in the Neurology Residency Program of the Santa Marcelina Hospital).; d Specialist in Neurological Rehabilitation at the Federal University of São Paulo, Specialist in Oral Motricity in Oncology Care at the A.C. Camargo Cancer Hospital (Speech and Hearing Therapist at the Santa Marcelina Hospital).; e MSc in Sciences, PhD in Medicine at the Federal University of São Paulo, Neurology and Neuroscience Program (Medical Supervisor of the Neurology Service and Coordinator of the Neurology Medical Residency Program at the Santa Marcelina Hospital).

**Keywords:** amyotrophic lateral sclerosis, deglutition, deglutition disorders, endoscopy, motor neuron disease

## Abstract

Amyotrophic lateral sclerosis (ALS) is a progressive degenerative motor neuron disease that adversely affects the muscles responsible for swallowing.

**Objective:**

To assess the oral preparatory, oral transit and pharyngeal phases of swallowing in ALS patients through endoscopic evaluation.

**Method:**

This cross-sectional historical cohort study included ALS patients submitted to endoscopic examination. Eleven patients (six males and five females; mean age of 61.7 years) were enrolled in the study from january to december of 2011.

**Results:**

All patients had alterations in phases of the swallowing process, but only 72.7% complained of dysphagia. The oral preparatory phase was altered in 63.6% of the subjects; the oral transit and pharyngeal phases were altered in all studied individuals, regardless of food consistency. Laryngeal penetration or tracheal aspiration were seen in 90.9% of the patients during the pharyngeal phase while they were swallowing fluids.

**Conclusion:**

Even in the absence of complaints, dysphagia is a frequent comorbidity in ALS patients. The oral transit and pharyngeal phases were the most frequently affected. Laryngeal penetration or tracheal aspiration occurred more frequently during the pharyngeal phase while patients were swallowing fluids.

## INTRODUCTION

Amyotrophic lateral sclerosis (ALS) is a rare disease with reported incidence rates ranging from 0.6 to 2.6 cases per 100,000 people^1^, ^2^, ^3^, ^4^. In 1830, Charles Bell, a famous British anatomist and surgeon, was the first to report on a condition that caused progressive paralysis of the limbs and tongue. French neurologist Jean-Martin Charcot was the first to describe ALS, also known as Charcot disease^1^. The condition gained notoriety after baseball player Lou Gehrig was diagnosed with it in 1939^1^.

Amyotrophic lateral sclerosis is a degenerative disease that affects the upper and lower motor neurons without causing sensory or cognitive impairment^3^. It is a progressive neurological disorder that produces degeneration of the motor system in various levels^2^, ^3^, ^4^. Practically any muscle group can be affected by this condition^5^. Dysphagia, alone or associated with dysarthria, is a common symptom in patients with ALS, particularly in subjects with bulbar involvement, whether it is due to degeneration of the lower motor neuron (bulbar palsy), upper motor neuron (pseudobulbar palsy), or both^2^, ^3^, ^4^, ^6^, ^7^.

Dysphagia, one of the most debilitating comorbidities to affect ALS patients, is caused by the progressive degeneration of the corticobulbar tract and/or the motor nuclei of cranial nerves IX, X, XI, and XII. It leads to secondary pharyngolaryngeal motility involvement and tongue atrophy and dyskinesia. The vagus and glossopharyngeal nerves innervate the pharynx and the larynx. The involvement of these cranial nerves affects the elevation of the soft palate and induces nasal regurgitation of food, reduced laryngeal elevation, and failure to protect the airways during deglutition^8^.

Patients with dysphagia associated with aspiration of food to the trachea may have aspiration pneumonia, a condition that requires hospitalization and the installation of alternative modes of nutrition. Prognosis is poorer for ALS patients with aspiration pneumonia. Early diagnosis of swallowing disorders may prevent such undesired developments and allow the establishment of an adequate course of treatment^9^, ^10^, ^11^.

Endoscopic examination provides information on the occurrence of stasis of salivary flow in the oropharynx, hypopharynx, and vestibule of the larynx, and allows the assessment of larynx and soft palate mobility. It can also be used to indirectly assess the oral preparatory, oral transit, and pharyngeal phases of swallowing by having the examined subjects swallow foods of different consistencies, in order to observe the presence of pharyngeal food stasis, oral food escape, and laryngeal penetration and/or tracheal aspiration^9^, ^12^, ^13^. One of the most significant advantages of endoscopic assessment of swallowing in progressive diseases is the possibility of assessing the sequence of events related to the disease's characteristic neurologic degeneration. This test provides results for foods of different consistencies and can be performed with the patient lying in bed and using nutritional or respiratory support, thus increasing its range in the diagnostic and follow-up settings^9^, ^12^.

The literature on ALS is extensive, but few authors have looked into the possibilities of endoscopic examination to assess deglutition. In this study, endoscopic evaluation of swallowing (EES) was used to indirectly assess the oral preparatory phase and directly analyze the oral transit and pharyngeal phases of deglutition in ALS patients. The tests were also used to correlate complaints of dysphagia and the verification of dysphagia in EES, and to assess the presence and occurrences of laryngeal penetration and tracheal aspiration.

### Casuistry

This study included all patients diagnosed with amyotrophic lateral sclerosis at the hospital's Neurology Clinic between January and December of 2011according to the criteria in the El Escorial^14^:

• Clinically definite ALS: evidence of upper and lower motor neuron dysfunction in three regions
• Clinically probable ALS: clinical evidence of lower and upper neuron dysfunction in at least two regions, with signs of upper motor neuron involvement at a more cranial level than the signs of dysfunction of the lower motor neurons
• Clinically probable ALS - laboratory supported ELA: clinical evidence of lower and upper motor neuron dysfunction in only one region and electromyographic evidence of lower motor neuron disorder in at least two limbs with lab workup and neurological imaging ruling out other possible diseases.

Subjects diagnosed with ALS, with and without signs or complaints of dysphagia, were sent for endoscopic examination at the hospital's ENT Clinic. Patients with other disorders that affected deglutition, in association or not with ALS, were excluded from the study.

Twelve patients underwent endoscopic examination, and one was excluded for having Parkinson's in association with ALS.

Five (45.5%) of the 11 subjects enrolled in the study were females and six (54.5%) were males. All patients had bulbar involvement, nine (81.8%) were fed orally, and two (18.2%) used nasoenteral feeding tubes. Patient mean age was 61.7 ± 7.2 years, the mean time since the onset of ALS symptoms was 26.0 ± 14.6 months, and the mean time since diagnosis was 13.9 ± 12 months.

## METHOD

This study was approved by the Research Ethics Committee and was granted permit 39/11. This is a historical cohort cross-sectional study carried out with amyotrophic lateral sclerosis patients.

Endoscopic examination was performed with a Pentax^®^ FLN10RP3 endoscope connected to a Watec 231s video camera, a Toshiba^®^ DVD recorder, a microphone, and a Sony^®^ 14-inch TV set. All tests were recorded on a DVD. The tests were carried out by the same examiner.

The subjects included in the study answered a questionnaire to assess deglutition disorders. They were examined for tongue mobility and fasciculations. Patients were seated during endoscopic examination. The flexible tip of the endoscope was inserted through the subject's nasal cavity without anesthesia or topical vasoconstrictors.

The foods provided to the subjects during endoscopic examination were dyed blue and offered in the following consistencies and volumes:

• Paste (spoon of 5 and 10 ml)
• Liquid (spoon of 5 and 10 ml)
• Solid (half a cracker).

The liquid used in the study was water. The paste was prepared with 100 ml of water and two spoons of thickener Resource Thicken up Nestle®. The solid food was half a cracker*.*

The following parameters were observed during examination:

• Posterior escape: progression of the food to the hypopharynx during the oral preparatory phase or after the pharyngeal phase of swallowing while food residues were present in the mouth
• Food stasis: presence of residual food in the piriform recesses and valleculae after three spontaneous deglutition attempts
• Laryngeal penetration: progression of the food to the glottis, without going beyond it
• Tracheal aspiration: progression of the food to under the vocal folds
• Time of occurrence of tracheal aspiration: in cases of present tracheal aspiration, the examiner checked whether it took place before the pharyngeal phase of swallowing (related to food posterior escape), and during or after the pharyngeal phase of swallowing (spillage of the food retained in the hypopharynx)
• Response to tracheal aspiration: presence of cough and/or hawking.

Alterations in the oral preparatory phase of swallowing were recorded when posterior food escape was observed; alterations in the oral transit phase were connected to presence of food residues in the valleculae; and alterations in the pharyngeal phase were associated with presence of food residues in the piriform recesses.

The results were analyzed through the test for equality of two proportions with a significance level of 0.05 (5%), a confidence interval of 95%, and a degree of freedom of one.

## RESULTS

All subjects included in the study had tongue fasciculations and bulbar involvement.

Eight (72.7%) patients reported dysphagia in the questionnaire.

All patients submitted to endoscopic evaluation of deglutition had swallowing phase dysfunctions.

The oral preparatory phase was altered in 63.6% of the subjects, and oral transit and pharyngeal phase dysfunctions were seen in all individuals, regardless of food consistency or use of a nasoenteral feeding tube.

Involvement of the oral and pharyngeal phases of swallowing were statistically significant (*p* < 0.05) for liquid, paste, and solid foods (Table 1).

**Table 1:** Alterations on each phase of swallowing for each type of food.

Liquid	No		Yes		*p*-value
	n	%	n	%	
Oral Preparatory Phase	6	54.5%	5	45.5%	670
Oral Transit Phase	0	0.0%	11	100%	< 0.001
Pharyngeal Phase	0	0.0%	11	100%	< 0.001

Paste					

Oral Preparatory Phase	7	63.6%	4	36.4%	201
Oral Transit Phase	1	9.1%	10	90.9%	< 0.001
Pharyngeal Phase	0	0.0%	11	100%	< 0.001

Solid					

Oral Preparatory Phase	5	50%	5	50%	1.000
Oral Transit Phase	1	10%	9	90%	< 0.001
Pharyngeal Phase	2	20%	8	80%	< 0.010

The pharyngeal phase of swallowing was compromised in all patients regardless of food consistency or use of a nasoenteral feeding tube.

The occurrence of laryngeal penetration or tracheal aspiration is described on Table 2. Laryngeal penetration or tracheal aspiration was seen in 10 (90.9%) patients during the pharyngeal phase of deglutition.

**Table 2:** Presence and time of occurrence of laryngeal penetration or tracheal aspiration.

Penetration or aspiration	Before laryngeal phase		During laryngeal phase		After laryngeal phase	
	n	%	n	%	n	%
Liquid	3	27.3%	10	90.9%	2	18.2%
Paste	1	9.1%	5	45.5%	1	9.1%
Solid	0	0.0%	2	18.2%	0	0.0%
Any consistency	3	27.3%	10	90.9%	2	18.2%

## DISCUSSION

All ALS patients enrolled in this study had swallowing phase dysfunctions, regardless of food consistency. The oral preparatory phase was altered in 63.6% of the subjects, and oral transit and pharyngeal phase dysfunctions were seen in all individuals. Re et al.^6^ used cineradiography to study the deglutition of 23 patients with ALS and reported oral transit phase alterations in 17 (73.9%) and pharyngeal phase alterations in 19 (82.6%) subjects.

Oral preparatory phase alterations in ALS patients stem from lower motor neuron impairment. The resulting weakness and lack of coordination of the face and tongue muscles may hamper the formation of the food bolus and the containment of food within the oral cavity^2^, ^8^, ^15^. However, oral preparatory phase alterations were not statistically significant in this study, possibly because endoscopic examination cannot be used to directly assess the preparation of the food bolus. Posterior food escape, seen in 63.6% of the studied patients, was observed through the subjects’ inability to contain the bolus in the oral cavity.

Tongue motor disorders appear to rank among the main factors contributing to dysphagia in ALS patients. In an indirect, however significant way, ejection force reduction negatively impacts the onset of the pharyngeal swallowing phase and the strength of the pharyngeal constrictor muscles, resulting in oropharyngeal dysphagia^2^. Tongue fasciculations were observed in all studied patients along with food residues in the pharynx - in the valleculae or piriform recesses, thus confirming the presence of oropharyngeal dysphagia.

Laryngeal penetration or tracheal aspiration of food was seen in 27.3% of the patients before the pharyngeal phase of swallowing, regardless of food consistency. This finding suggests the patients were at risk for not being able to contain the food bolus in the oral cavity. Ertekin et al.^10^ analyzed the electromyograms of the submental and cricopharyngeal muscles of 43 subjects diagnosed with ALS and observed reduced control of the tongue and submental muscles and abnormal laryngeal elevation during the pharyngeal phase of deglutition. These findings may account for posterior escape of food during the oral preparatory phase, culminating with tracheal aspiration before the pharyngeal phase of swallowing. According to a meta-analysis carried out by Pontes et al.^2^, early food escape is more frequent with thin liquids and ranks as the top cause for tracheal aspiration, even in the early stages of the disease and mild oral muscle alteration. In this study, liquid foods had the highest risk for laryngeal penetration or tracheal aspiration. However, these alterations were seen in 90.9% of the patients during the pharyngeal phase of swallowing.

Food stasis in the hypopharynx was seen in 100% of the subjects while they were having liquid and paste foods, and in 81.8% of the cases when they were fed crackers. Ertekin et al.^10^ correlated the retention of food in the pharynx with cricopharyngeal muscle hypertonicity and lack of coordination between this muscle and the laryngeal elevators during deglutition, facilitating tracheal aspiration of the food retained in the pharyngeal space as it went down the larynx. Most (90.9%) laryngeal penetration or tracheal aspiration events observed in this study took place during the pharyngeal phase of swallowing; 18.2% occurred after the pharyngeal phase of deglutition.

Re et al.^6^ used cineradiography to study the deglutition of 23 patients with ALS and reported laryngeal penetration in 14 (60.9%) cases, one of which before, five during, and eight after the pharyngeal phase of swallowing. The authors also found five (21.7%) cases of tracheal aspiration during the pharyngeal phase of deglutition. Reduced hyoid and larynx elevation and impaired pharyngeal constrictor muscle contraction were the explanations given for laryngeal penetration or tracheal aspiration during the pharyngeal phase of swallowing. Laryngeal penetration of tracheal aspiration after the pharyngeal phase of deglutition was attributed to food stasis in the valleculae and piriform recesses^6^.

Although amyotrophic lateral sclerosis has been classically considered as a motor neuron disease, some authors have described associations between ALS and reduced pharyngolaryngeal sensation. Amin et al.^16^ analyzed 22 patients with ALS for laryngeal sensation through nasal endoscopy and verified that 54.5% of the subjects had altered test results. If sensation alterations in ALS patients are indeed associated with reduced motor response, patients should not be expected to hawk, cough, or choke during deglutition to confirm the presence of dysphagia in ALS.

In our study, even though only 72.7% of the patients complained of dysphagia, all had some form of swallowing alteration in the endoscopic examination, making it mandatory for dysphagia to be investigated in cases of amyotrophic lateral sclerosis. Endoscopy is a low-cost widely available examination that can be used routinely to assess ALS patients to avoid the complications related to the deglutition disorders seen in this population.

## CONCLUSION

The endoscopic evaluation of swallowing of patients with amyotrophic lateral sclerosis revealed that, even in the absence of complaints, dysphagia was a frequent comorbidity. The oral transit and pharyngeal phases of deglutition were the most frequently involved, regardless of food consistency. In this study, laryngeal penetration or tracheal aspiration occurred more frequently during the pharyngeal phase of swallowing, with liquid foods presenting the strongest correlation with these alterations.
